# Outcome after microvascular decompression for trigeminal neuralgia in a single center—relation to sex and severity of neurovascular conflict

**DOI:** 10.1007/s00701-023-05642-2

**Published:** 2023-06-07

**Authors:** Richard Loayza, Johan Wikström, Anna Grabowska, Robert Semnic, Hans Ericson, Sami Abu Hamdeh

**Affiliations:** 1 Department of Medical Sciences, Section of Neurosurgery, Uppsala University, Uppsala, 751 85 Sweden; 2 Department of Surgical Sciences/Section of Neuroradiology, Uppsala University Hospital, Uppsala University, Uppsala, 751 85 Sweden

**Keywords:** Trigeminal neuralgia, Microvascular decompression, Facial pain, Neurovascular conflict

## Abstract

**Background:**

Trigeminal neuralgia (TN), a severe type of facial pain, is mainly caused by a neurovascular conflict (NVC). The severity of the NVC seems associated with the outcome following microvascular decompression (MVD) surgery. This study aimed to investigate the outcome after MVD and whether it is affected by NVC severity and sex.

**Methods:**

TN patients (*n* = 109) were followed for 5 to 10 years after MVD. Barrow Neurology Index (BNI), Patients Global Impression of Change (PGIC), complications, and time to relapse were evaluated. The NVC severity was retrospectively reviewed from presurgical MRI. Demographic and clinical factors and NVC severity were analyzed for potential association with outcome after MVD.

**Results:**

The success rate (BNI ≤ 2) was 80% after 5 to 10 years follow-up for TN patients with severe NVC (grade 2–3) and 56% for TN patients with mild NVC (grade 0–1, *P* = 0.003). No sex difference was observed in outcome for patients with both mild (*P* = 0.924) and severe NVC (*P* = 0.883) respectively. Three patients (2.8%) during the hospital stay, and two patients (1.8%) at 6 weeks, experienced a complication requiring invasive treatment. At long-term 52/109 patients (47.7%) reported some type of persistent adverse event, of which the majority were mild and required no treatment.

**Conclusions:**

MVD offers an 80% probability of long-term pain relief in TN patients with severe NVC, with low frequency of serious complications. NVC severity significantly affects outcome after MVD, while no sex differences in outcome were found. In consistency with previous work, the results stress the importance of adequate neuroradiological assessment of the NVC for preoperative patient selection.

## Introduction

Trigeminal neuralgia (TN) is characterized by excruciating facial pain, in the distribution of the trigeminal nerve, which is typically triggered by harmless stimuli [10]. The pain is stabbing, intense, cutting and comes in short intervals. Classical trigeminal neuralgia is primarily caused by a blood vessel, commonly the superior cerebellar artery (SCA), compressing the nerve at the trigeminal root entry zone (TREZ) near the brainstem, without other associated conditions. Nonetheless, a neurovascular conflict (NVC) does not explain all cases of TN, and TN can exist without an NVC [17, 24].

First line treatment is carbamazepine and/or oxcarbazepine which offers pain relief in 60–70% of patients [5]. Surgical treatment, with microvascular decompression (MVD) of the offending vessel is second line treatment and may offer excellent outcomes in about 70% of patients [28]. The complication rate is low (< 13.3%), where facial numbness and unilateral hearing loss are the most common [2, 4, 23]. The timing of surgery is however still under debate [8].

TN is more prevalent in females, with a ratio of 1:1.5–1:1.7 and this may be due to longer longevity [12, 18, 28]. The impact of sex on the outcome of MVD surgery is still under debate, where increased frequency of pain relapse in women after MVD has been shown in some studies [1, 13, 22] but not in others [9, 21, 27]. Furthermore the presence and severity of NVC have been shown to be predictors of the success of MVD [19, 20], but some patients with mild NVC may still benefit from surgery [20].

The aim of this study was to analyze the outcome and complication rate after MVD in our center and to investigate the role of sex and NVC severity on the success rate of MVD surgery.

## Methods

### Patients

Patients that fulfilled the criteria for classical TN according to ICDH-3 beta 2013 [27] and had undergone MVD for TN from December 2009 to April 2018 at the Department of Neurosurgery at the University Hospital in Uppsala, Sweden, were eligible for this retrospective cohort study. The MVD surgeries were performed by two alternating neurosurgeons (HE and SAH), specialized in this area, via a retrosigmoid approach. A total of 167 operations were performed during the study period. Patients were contacted by phone for informed consent to participate in the study after being given the opportunity to ask questions. The patients who agreed to participate in the study and did not fulfill any exclusion criteria were included. The exclusion criteria were as followed.

### Exclusion criteria

- Not contactable or did not agree to participate
- Passed away before the follow-up
- Not in mental condition to give relevant answers when contacted
- Non-existing and/or non-evaluable pre-surgical MRI

### Imaging and image analysis

MR imaging was performed at different hospitals in the Uppsala Region, and the imaging protocol hence varied somewhat. Evaluation of neurovascular conflict was performed on high-resolution fluid-sensitive sequences (CISS, DRIVE, or similar, depending on manufacturer).

Three neuroradiologists, with varying degree of experience, assessed the patients' MRI examinations. The images were judged independently and blinded to the side of the TN. The assessment included the following;

- Extent of contact between the trigeminal trunk and a blood vessel:

- Grade 0: No contact between nearby blood vessels and the nerve.
- Grade I: The blood vessel is in contact with the nerve without causing any displacement or impression.
- Grade II: The blood vessel displaces or deforms the nerve.
- Grade III: The blood vessel causes a pronounced impression in the nerve.

- The identity of the offending vessel.

The degree of the NVC, was dichotomized in mild NVC including grades 0 and 1 and severe NVC including grades 2 and 3 for statistical analysis. The initial assessment was made by the first two neuroradiologist (agreement rate = 56.7%, Cohen’s kappa = 0.199). Disagreement was settled by the assessment of a senior neuroradiologist (JW) with expertise in the field.

### Short-term follow-up

Patients were followed up by the operating surgeons postoperatively and at 6 weeks. The outcome after MVD surgery was assessed using the scale from the Barrow Neurological Institute (BNI) [11]. From these follow-ups, data was collected from the patients’ journals regarding length of stay, reported complications and BNI as well as reoperations with a new MVD or balloon compression, if they had occurred.

### Complications

Complications were defined as adverse event occurring within 6 weeks from surgery and classified using the classification for neurosurgical complications proposed by Ibañez et. al. [11], focused on a four-level severity grading system as follows;

- Grade I: Any non–life-threatening deviation from normal postoperative course, not requiring invasive treatment

- Grade Ia: Complication requiring no drug treatment
- Grade Ib: Complication requiring drug treatment

- Grade II: Complication requiring invasive treatment such as surgical, endoscopic, or endovascular interventions

- Grade IIa: Complication requiring intervention without general anesthesia
- Grade IIb: Complication requiring intervention with general anesthesia

- Grade III: Life-threatening complications requiring management in ICU

- Grade IIIa: Complication involving single organ failure
- Grade IIIb: Complication involving multiple organ failure

- Grade IV: Complication resulting in death

Each complication was graded separately and during the stay at the neurosurgical ward and for the 6 weeks follow-up.

### Long-term follow-up

Telephone interviews were performed for outcome assessment at long-term (range 60 to 117 months). Patients who did not respond were sent a text message if a mobile phone was available. The others were called again 2–3 business days after the first trial, in up to three trials. Patients who did not respond to calls or text messages were excluded from the study. The outcome after MVD surgery was assessed using BNI [11] and Patients Global Impression of Change (PGIC [1, 25]. The patient was finally asked whether there were any remaining adverse events since the surgery. Success of MVD surgery was defined as BNI ≤ 2.

## Results

### Patient population

**Fig. 1:**
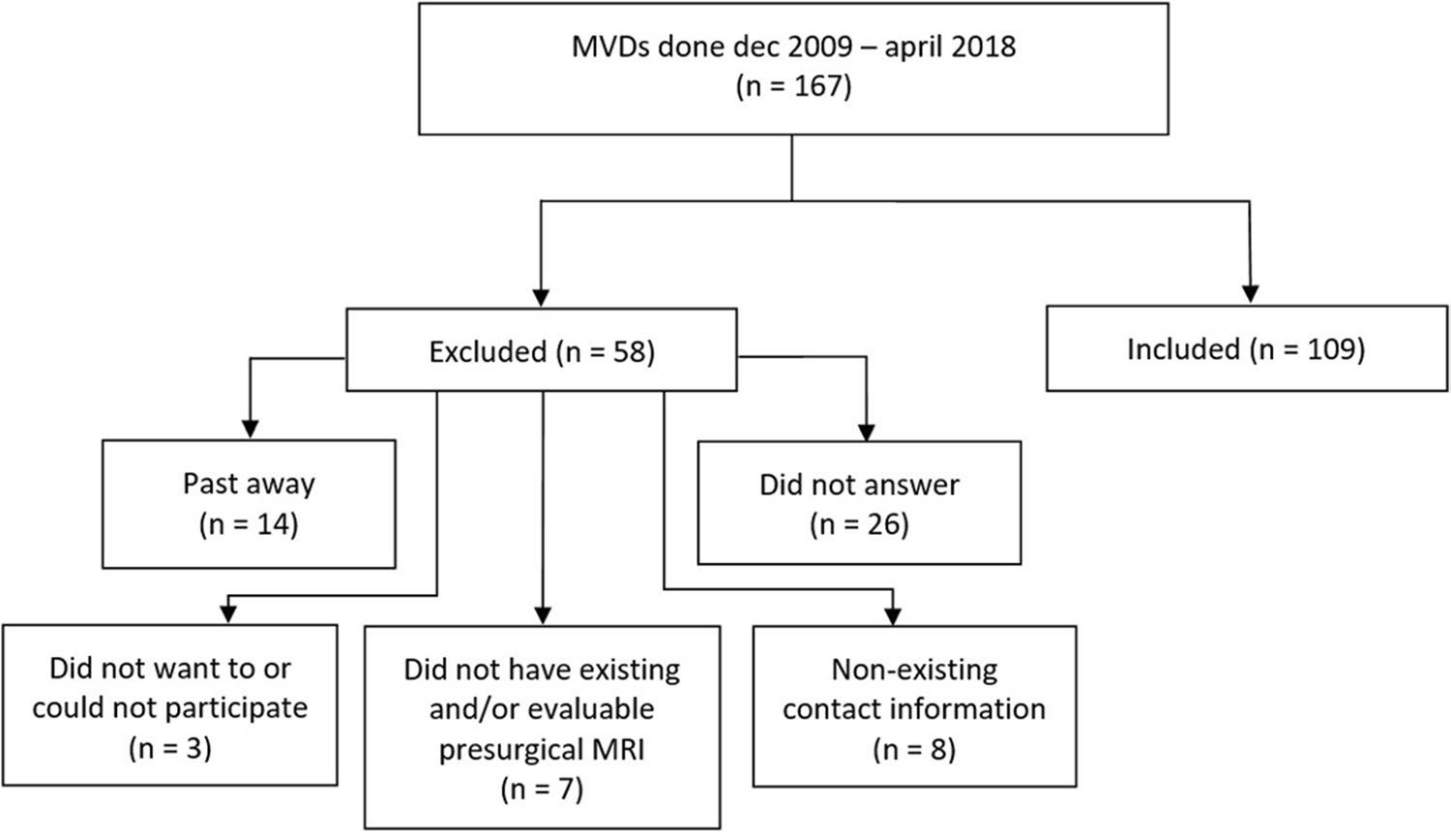
Flow diagram of the patient population included in the study

From a total of 167 patients meeting the inclusion criteria 26 did not answer, 14 had passed away, eight did not have any existing contact information, seven did not have any existing and/or evaluable presurgical MRI and three did not agree to participate. Thus, 58 patients were excluded, and 109 patients were included in this study (Fig. 1). The excluded patients did not differ in age, sex, BNI, frequency of relapse or resurgery at 6 months follow up (*P* > 0.05).

Demographic and clinical data are shown in Table 1. Mean age of the TN patients was 62 years (range 23–87 years, 53 males, 56 females). The preoperative MRI depicted a severe NVC in 48 (44%) and a mild NVC in 61 (56%) of the TN patients respectively. The SCA was the offending vessel in most patients (*n* = 62, 56.9%). Venous NVCs were of mild severity more frequently than arterial NVCs (*P* < 0.001). There were no differences between the sexes in the proportions of arterial vs venous NVC (*P* = 0.46).

**Table 1:** Demographic, clinical and outcome data of trigeminal neuralgia patients

Variable	No of cases	Percent (%)
Sex (male/female)	53/56	48.6%/51.4%
Age (years)†	109	62, range 23–87
NVC grade		
Mild (0–1)	61	56.0%
Severe (2–3)	48	44.0%
Offending vessel		
SCA	62	56.9%
AICA	4	3.7%
Vertebral artery	1	0.9%
PICA	1	0.9%
Basilar artery	2	1.8%
Unknown artery	2	1.8%
Vein	24	22.0%
Unknown vessel	13	11.9%
Length of stay (days) †	109	4, range 1–17
Follow-up time (months) †	109	85, range 60–117
BNI 6 weeks		
1	74	67.9%
2	5	4.6%
3	8	7.3%
4	2	1.8%
5	0	0.0%
N/A	20	17.4%
BNI long-term		
1	69	63.3%
2	15	13.8%
3	10	9.2%
4	13	11.9%
5	2	1.8%
N/A	0	0.0%
PGIC long-term		
1	84	77.1%
2	15	13.8%
3	4	3.7%
4	3	2.8%
5	0	0.0%
6	2	1.8%
7	1	0.9%
Pain relapse long-term		
Yes	35	32.1%
No	74	67.9%

### Outcome of MVD surgery measured with Barrow Neurological Institute (BNI) scale

Short- and long-term outcomes are presented in Table 1. At the long-term follow-up, TN patients with a severe NVC had a better outcome after MVD, with a significantly lower BNI (median 1, range 1–4) than those with a mild NVC (median 1, range 1–5), regardless of sex (male *P* = 0.035, female *P* = 0.025, both sexes *P* = 0.002). No statistically significant differences in reported BNI at long-term was found between the sexes, neither in total (male median 1, range 1–5, female median 1, range 1–5, *P* = 0.585), nor when males and females within the two NVC severity groups were compared (mild NVC *P* = 0.924; severe NVC *P* = 0.883).

### Outcome of MVD surgery measured with Patients Global Impression of Change (PGIC)

At the long-term follow up, TN patients with a severe NVC had a better outcome after MVD, with a significantly lower PGIC (median 1, range 1–2) than those with a mild NVC (median 1, range 1–7), regardless of sex (male *P* = 0.013; female *P* = 0.045; both sexes *P* = 0.001). No significant difference in reported PGIC at long-term was found between the sexes in total (male median 1, range 1–6, female median 1, range 1–7,* P* = 0.070), nor when males and females within the two NVC severity groups were compared (mild NVC *P* = 0.449; severe NVC *P* = 0.162).

### Success rate of microvascular decompression at long-term follow up

**Fig. 2:**
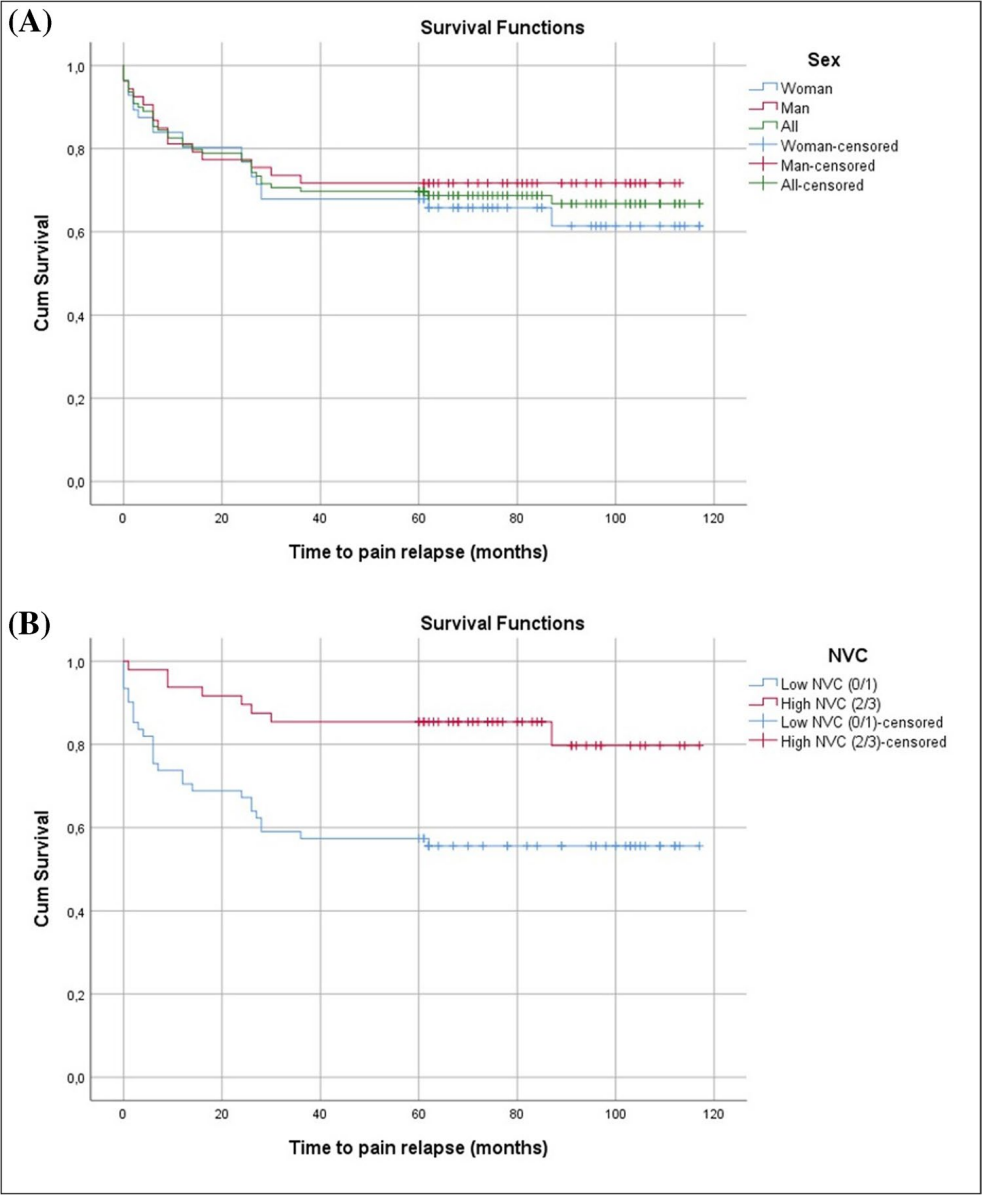
(**A**) Kaplan Meier curve showing time to relapse in males and females, respectively, and (**B**) in patients with mild and severe NVC, respectively. There was no statistically significant difference between the sexes (Cox regression, HR = 1.3, *P* = 0.444). Patients with mild NVC had a significantly worse outcome than patients with a severe NVC (Cox regression HR = 3.247, *P* = 0.003)

The total success rate for MVD was 66.8% after 5–10 years follow-up. Cox regression did not show any difference in the success rate for MVD in females (61.4%) when compared to males (71.7%), (Cox regression, HR = 1.3, *P* = 0.444; Fig. 2). TN patients with mild NVC had a lower success rate (55.6%) for MVD than TN patients with severe NVC (79.7%), (Cox regression HR = 3.247, *P* = 0.003; Fig. 2).

### Complications

During the postoperative period, before dismissal from the neurosurgical department, 28 patients (25.7%) experienced a complication of which 20 (18.3%) did not require treatment (1a) (Table 2). The remaining eight patients (7.4%) included three CSF leakages (2.8%), two lower urinary tract infections (1.8%), one urosepsis (0.9%), one deep vein thrombosis (0.9%) and one pancreatitis (0.9%).

**Table 2:** Complications according to the follow-up checkpoints

Variable	No of cases	Percent (%)
Complications during stay at the neurosurgical department		
0	81	74.3%
1a	20	18.3%
1b	5	4.6%
2a	0	0.0%
2b	3	2.8%
3a	0	0.0%
3b	0	0.0%
4	0	0.0%
Complications after 6 weeks		
0	85	78.0%
1a	17	15.6%
1b	5	4.6%
2a	0	0.0%
2b	2	1.8%
3a	0	0.0%
3b	0	0.0%
4	0	0.0%
Persistent adverse events at long-term		
No adverse event	57	52.2%
One adverse event	38	34.9%
Two adverse events	14	12.8%
Discomfort in the scar	23	21.1%
Some loss of facial sensation	33	30.3%
Impact on balance	5	4.6%
Impact on hearing	1	0.9%
Chewing problems	2	1.8%
Stroke	2	1.8%

At the 6 weeks follow-up, 24 patients (22.0%) reported complications of which 17 (15.6%) did not require treatment (1a) (Table 2). The remaining seven patients (6.4%) included two atrial fibrillations (1.8%), two wound infections (1.8%), one urosepsis (0.9%), one CSF leakage (0.9%) and one stroke (0.9%).

At the long-term follow-up, 52 patients (47.7%) reported any type of persistent adverse event, the two most common being some loss of facial sensation (30.3%) and discomfort in the scar (21.1%) (Table 2). There were no significant associations between persistent adverse events and age, sex, degree of NVC or arterial vs venous compression (*P* > 0.05).

## Discussion

In this study, we evaluated the outcomes after 109 MVDs for TN in a single center. The severity of the NVC was significantly associated with the surgical outcome. After 5–10 years, the success rate was 80% in patients with severe NVC and 56% in patients with mild NVC, respectively. These results are in concordance with previously published results [1, 3, 7, 20, 25, 29]. The criteria for a successful outcome in previous studies vary, although they are comparable to the criteria in the present report. Barker et al. [1], included 1204 patients in the largest study conducted to date and found a success rate of 69.6% after 10 years, categorized into three groups, excellent (> 98% pain relief, no medication), good (> 75% pain relief, intermittent medication was allowed) and failure (> 25% of preoperative pain level). Sindou et al. [20] did a similar study in which the NVC was graded in the severities 1 to 3 and observed a success rate of 83.3% for grade 1, 90.2% for grade 2 and 96.6% for grade 3 at 1 year follow up and 58.3% for grade 1, 78.3% for grade 2 and 88.1% for grade 3 at 15 years follow up. Wang et al. [26] used the BNI scale and found a success rate of 85, 61% and 44% after 1, 5, and 10 years, respectively. In addition to BNI, we used PGIC to evaluate the patient’s own impression of pain relief after the surgery. The PGIC showed a significant improvement (PGIC ≤ 3; 95.4%) after MVD.

The radiologist’s assessment and experience are of importance when evaluating the NVC severity, as it can impact the prediction of MVD outcome. In this study, two neuroradiologists assessed the preoperative MRI and there was only a fair agreement between the two raters, plausibly due to differences in experience and in the MRI quality. Disagreement was settled by the reassessment of the images by a third senior neuroradiologist, minimizing the risk of misjudgment. Assessment of NVC severity was performed using high-resolution fluid-sensitive sequences, which are reliable for detecting NVC [14]. Nevertheless, the association between NVC severity and outcome enforces the value of high-quality preoperative MRI and experienced neuroradiologists in selecting appropriate patients for MVD. Although MRI is reliable in predicting high-grade neurovascular compression, low-grade compressions on MRI may still be revealed as false positives during MVD surgery [6]. Additional measurements to evaluate atrophic changes such as volume and cross-sectional area of the trigeminal nerve may enhance prognostication [15].

Some studies have shown that women are more prone to relapse after MVD for TN than men [1, 13, 22], although it has been contradicted in others [9, 21, 27]. The propensity of relapse in females may be due to a preponderance of venous NVC, where the recurrence rate for TN is greater [16]. Although venous NVCs were of mild severity more frequently than arterial NVCs in our study, we could not observe such an association.

In this study, we used Ibañez grading system [11] to assess short-term complications up to 6 weeks post-surgery. The most common complications were in groups “1a” and “1b” although a few in group “2b” requiring surgical intervention. A meta-analysis showed a total perioperative complication rate of 13.3% where facial numbness (4.4%) was the most common followed by unilateral hearing loss (1.8%). Additionally, major complications such as CSF leakage (1.3%) and meningitis (1.2%) were noted [4]. Major complications of 1.2–5.2% were also reported by Bartek Jr. et al. [2] and by Theodros et al. with a complication rate of 6.5% and mortality rate of 0% [23].

At long-term, some patients had one or two persistent complains, the most common being loss of facial sensation or discomfort in the scar. Major persistent complications were rare, with two strokes occurring during the follow-up period, one of which had a long-term impact on motor function. Thus, MVD is a safe and effective treatment for TN in the presence of a significant NVC, with most complications being mild and not affecting daily life.

This study is limited by its retrospective design and the relatively small sample size of a single center cohort. Nonetheless, all patients were operated by the same two surgeons using the same surgical technique. Additionally, follow-up at our department is standardized for patients operated with MVD causing few patients to be lost to follow up.

## Conclusion

We conclude that a severe NVC correlates with a successful outcome (BNI ≤ 2) and an improved patient impression of change (PGIC ≤ 3) in the treatment of TN with MVD. Sex did not correlate with success of MVD surgery in TN patients, regardless of the NVC severity. Complications after MVD surgery are mostly mild and tolerable for the patient at long-term. In consistency with previous work, the results stress the importance of adequate radiological assessment of the NVC for preoperative patient selection.

## Data Availability

The data used in this study is available upon reasonable request.
